# The value of urinary interleukin-18 in predicting acute kidney injury: a systematic review and meta-analysis

**DOI:** 10.1080/0886022X.2022.2133728

**Published:** 2022-10-19

**Authors:** Zheng Qin, Hancong Li, Pengcheng Jiao, Luojia Jiang, Jiwen Geng, Qinbo Yang, Ruoxi Liao, Baihai Su

**Affiliations:** a Department of Nephrology, National Clinical Research Center for Geriatrics, West China Hospital of Sichuan University, Chengdu, China; b Med-X Center for Materials, Sichuan University, Chengdu, China; c Med + Biomaterial Institute of West China Hospital/West China School of Medicine of Sichuan University, Chengdu, China; d West China School of Medicine, West China Hospital of Sichuan University, Chengdu, China

**Keywords:** Interleukin-18, acute kidney injury, systematic review, meta-analysis

## Abstract

**Aims:**

The aim of this study was to systematically review relevant studies to evaluate the value of urinary interleukin-18 (uIL-18) in predicting acute kidney injury (AKI).

**Methods:**

A comprehensive search of PubMed, Medline, Embase, and Cochrane Library was conducted for literature published up to 1 August 2022. Quality Assessment Tool for Diagnostic Accuracy Studies-2 (QUADAS-2) was applied to assess the literature quality. Then, relevant data were extracted from each eligible study and a random-effects regression model was utilized to pool sensitivity, specificity, and construct summary receiver operating characteristic (SROC) and area under curve (AUC).

**Results:**

Twenty-six studies with 7183 patients were enrolled and relevant information was extracted. The estimated sensitivity and specificity of uIL-18 in the diagnosis of AKI were 0.64 (95% confidence interval (CI): 0.54–0.73) and 0.77 (95%CI: 0.71–0.83), respectively. The pooled diagnostic odds ratio (DOR) was 6.08 (95%CI: 3.63–10.18), and the AUC of uIL-18 in predicting AKI was 0.78 (95%CI: 0.74–0.81). Subgroup analysis showed that uIL-18 in pediatric patients was more effective in predicting AKI than in adults (DOR: 7.33 versus 5.75; AUC: 0.81 versus 0.77).

**Conclusions:**

Urinary IL-18 could be a relatively good biomarker with moderate predictive value for AKI, especially in pediatric patients. However, further research and clinical settings are still needed to validate our findings.

## 1. Introduction

Acute kidney injury (AKI) is mainly manifested by the rapid decline of renal function in a short period of time and the accumulation of metabolic waste with a high morbidity and mortality [1,2]. It is reported that the incidence of AKI was 21.6% and the mortality rate related to AKI was 23.9% in adults [3]. A multicenter epidemiological study showed that the prevalence of AKI was 26.9% in critically ill children [4]. The early diagnosis and intervention for AKI not only provides better treatment options, but also improves patient prognosis [5].

At present, increased serum creatinine (SCr) and decreased urine volume are the main clinical indicators of AKI. However, due to the influence of muscle mass, diuretics and other factors, the changes of SCr and urine volume were unstable and lagged [6,7]. Both SCr and urine volume have limitations in the timely and accurate identification of decreased renal function, which may delay the diagnosis of AKI thus leading to the poor prognosis. The increase of SCr is mainly caused by the decrease of glomerular filtration rate (GFR) and usually could be observed until 2–3 days after the AKI occurrence [8]. All these facts demonstrated the urgent need for more effective predicting of AKI, and an increasing number of studies have been conducted in order to provide more information about this topic [9]. Several new biomarkers in serum and urine have been reported to show the potential for predicting AKI, including cystatin C (Cys C), neutrophil gelatinase-associated lipocalin (NGAL), interleukin-18 (IL-18), kidney injury molecular-1 (KIM-1), and so on [10,11]. Among them, IL-18 has aroused widespread attention. IL-18 is a member of the IL-1 family of cytokines, which is synthesized as an inactive 23 kDa precursor by monocytes, macrophages, and proximal renal tubular epithelial cells. In animal models, IL-18 could be activated by caspase-1, thus inducing ischemic injury and inflammation in the proximal renal tubule, then excreted into urine [12,13]. Previous studies about urinary IL-18 (uIL-18) in predicting AKI suggested that uIL-18 may act as a better indicator of decreased renal function compared with SCr [14–17]. Its expression was upregulated in the early stage of AKI and could be detected well earlier than SCr, thus showing its potential in predicting AKI [18,19]. Although an increasing number of related studies have been conducted recently, additional clinical research and trials are still needed to validate the application of uIL-18 in different clinical settings [20].

With the accumulation of evidence, conflicting results have raised concerns about the predictive value of uIL-18 for AKI. To further clarify the performance and clinical detection value of uIL-18, we conducted a systematic review and meta-analysis based on 24 original articles, which help to evaluate its role in the early detection of AKI.

## 2. Methods

### 2.1. Data Source and search strategy

This meta-analysis was performed according to the Preferred Reporting Items for Systematic Reviews and Meta-Analyses (PRISMA) statement [21]. A comprehensive search of PubMed, Medline, Embase, and Cochrane Library was conducted to identify relevant articles published up to 1 August 2022. The search strategy was applied to identify all literatures with following keywords: ‘IL-18’ or ‘interleukin 18’ plus ‘acute kidney injury’ or ‘acute renal failure’ or ‘AKI’ or ‘ARF’. The details of search methods have been provided in Supplemental Item 1. In addition, we also checked the references of relevant studies and reviews manually to identify other potentially eligible studies. The search was preformed independently by two investigators (ZQ and HL).

### 2.2. Study selection

We encompassed all articles and conference papers retrieved without language or sample size restrictions. Studies were assessed and selected based on titles and abstracts by two independent reviewers (ZQ and HL) using the Endnote bibliography manager blinded to the authors and institute of studies and then retrieved and rescreened full-text articles. Further, the citations of reviewed full-text articles were also checked to avoid missing additional relevant studies. Any conflicts were resolved by a third reviewer (JG, blinded to the authors and institute of studies). The exclusion criteria were as follows: (1) review articles or duplicate papers. (2) Animal or *in vitro* based studies. (3) Studies could not provide the diagnosis value of uIL-18. Although there were no language restrictions initially, for the full-text review and data extraction, only articles in English language were included for the further analysis.

### 2.3. Data extraction and quality assessment

After a detailed full-text review of each study, two independent reviewers (ZQ and JP) utilized a standardized form to extract data from the retained studies. Disagreements were resolved by discussion and assessed by a third reviewer (PJ) until a consensus was reached. Extracted data were as follows: (1) research information: first author, year of publication, original country, sample size, study design, and population setting; (2) patient characteristics: age, sex, and baseline IL-18; (3) information of AKI: definition of AKI and number of patients who developed AKI; (4) information of IL-18: the time of obtaining specimen, assay method, and sample storage; (5) cutoff value for urinary IL-18, specificity, sensitivity, and area under the ROC curve (AUC) with 95% confidence interval (CI). The true positive (TP), true negative (TN), false positive (FP), and false negative (FN) results were calculated. If there is more than one cutoff value in a study, the cutoff value with the highest product of specificity and sensitivity was used.

Quality assessment was performed by two independent reviews (ZQ and HL) using the Quality Assessment Tool for Diagnostic Accuracy Studies-2 (QUADAS-2) [22]. Any discrepancies were resolved by a third reviewer (PJ).

### 2.4. Statistical analysis

All statistical analysis was performed by STATA version 14.0 (Stata Corp., College Station, TX) using the MIDAS module [23]. The kappa statistic was utilized to evaluate the agreement between two investigators in study selection. A bivariate random-effects regression model was used to calculate the pooled sensitivity (SEN), pooled specificity (SPE), the positive likelihood ratio (PLR), the negative likelihood ratio (NLR), and the diagnostic odds ratio (DOR) with their 95% CIs based on DerSimonian–Laird method [24]. A hierarchical summary ROC curve was constructed and the area under the curve was calculated to assess the overall diagnostic accuracy [25]. Deek’s funnel plots were constructed to evaluate the publication bias and *p* < 0.1 was believed representative of statistically significant publication bias. The *I*^2^ index was calculated to detect the heterogeneity between studies. The value of *I*^2^ lied between 0% and 100%, a value of 0% suggested no observed heterogeneity, and *I*^2^ values above 50% were regarded as indicative of substantial heterogeneity. Meta-regression was used to explore the source of heterogeneity. In addition, we conducted a subgroup analysis according to patients age (adults or pediatrics), predictive time (≤12 h or >12 h), the time of obtaining specimen (admission or other times) and the population setting (cardiac surgery patients or others), publication date (before or after 2009), AKI definition (using standardized definition or not) and population continents (Asia, North America, and others) to explore the potential sources of heterogeneity between included studies.

## 3. Results

### 3.1. Search results and study characteristics

**Figure 1.**
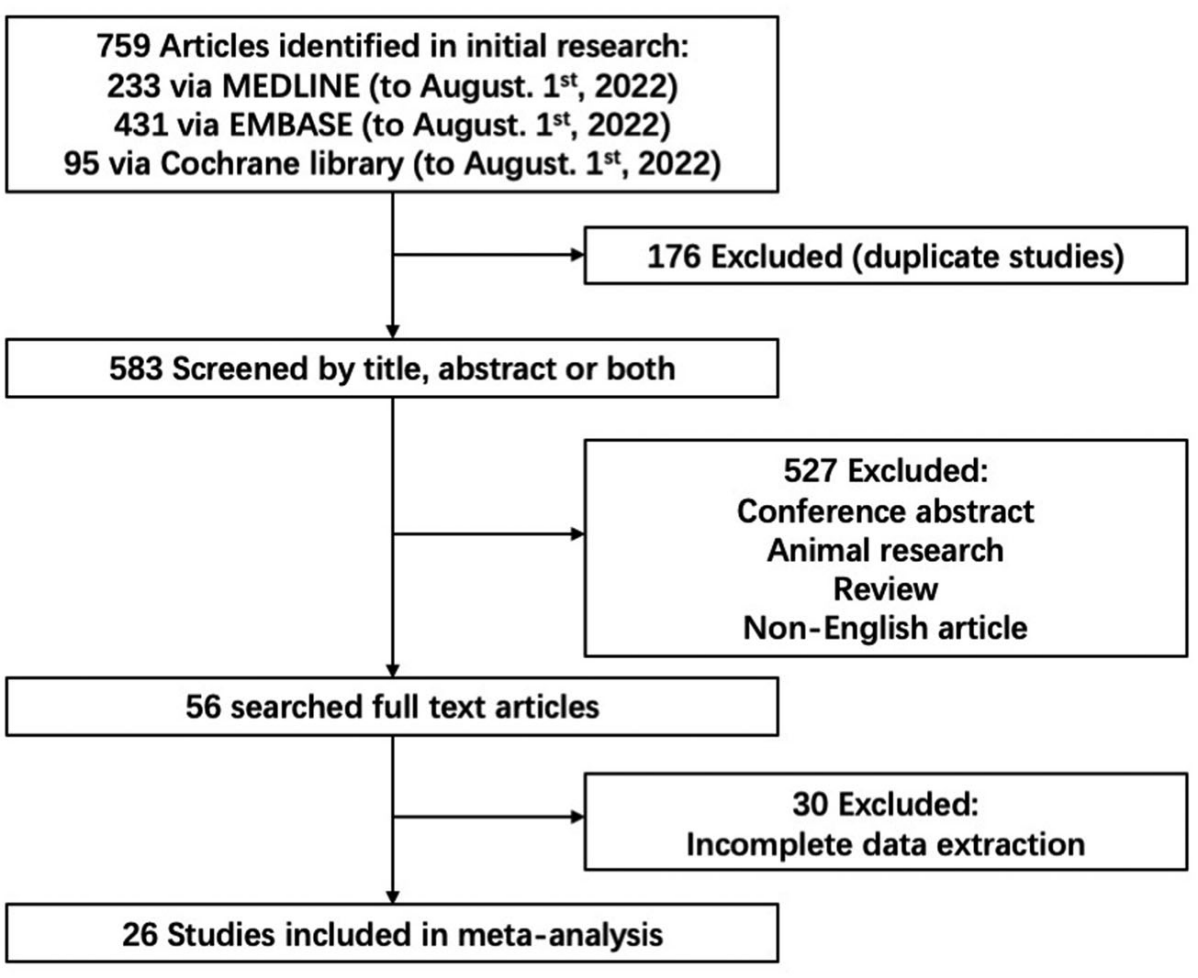
Flowchart for the review process and outcomes of inclusion and exclusion.

A total of 759 publications from different databases were retrieved upon the initial search. First, 176 studies were excluded after duplicates were identified. Then, we screened the titles and abstracts of the remaining 583 articles. Five hundred and twenty-seven of them were excluded because they are conference abstract, animal research, review, or non-English article. There were 56 studies selected for full-text review, and 30 studies were removed due to the incomplete data extraction. The kappa statistic following abstract review was 0.82 (*p* < 0.05), indicating substantial agreement between reviewers. The details of data for kappa statistic are shown in Supplemental Table 1. Finally, 26 studies with 7183 patients were included in this meta-analysis [20,26–50] (Figure 1).

The characteristics of included studies are shown in Table 1. All these studies were published from 2005 to 2021, varied in country, study design, sample size (from 40 to 1493), and population settings. It is worth noting that these studies defined AKI differently. In these 26 studies, 21 studies focused on adults, and the left five studies focused on the pediatric patients (age <18 years old). Twelve studies were conducted on patients who underwent cardiac surgery, other population settings included intense care unit patients, burn patients, non-septic critically ill neonates, and so on. All of studies determined IL-18 concentration by enzyme-linked immunosorbent assay (ELISA) and the samples were stored at −80 °C.

**Table 1.** Characteristics of studies included in the meta-analysis.

Study	Country	*N*	Patients with AKI (%)	Population setting	Mean age (years)	Males (%)	Mean baseline IL-18 (pg/ml)	Definition of AKI	IL-18 assay	Sample storage (°C)
Parikh et al. [26]	USA	138	52 (37.7)	ARDS and ALI adults	50.2 ± 17.0	72 (52.2)	AKI: 104 (0–955) Non-AKI: 0 (0–173)	SCr↑ ≥50% baseline	ELISA	–80
Parikh et al. [20]	USA	55	20 (36.4)	Cardiac surgery children	3.4 ± 5.3	30 (54.5)	1.65 ± 1.01	SCr↑ ≥50% baseline	ELISA	–80
Washburn et al. [27]	USA	137	103 (75.2)	PICU children who received mechanical ventilation	6.5 ± 6.4	73 (53)	179.0 ± 337.9	Pediatric modified RIFLE	ELISA	–80
Ling et al. [48]	China	40	13 (8.7)	CIN adults	AKI: 66.38 ± 9.9; non-AKI: 68.62 ± 10.6	24 (60)	AKI: 12.06 (10.79–17.17); non-AKI: 10.57 (7.88–13.86)	SCr ≥ 0.5 mg/dl or ↑≥25% within 48–72 h	ELISA	–80
Haase et al. [44]	Australia	100	20 (80)	Adults went through CPB	AKI: 75.3 ± 4.1 Non-AKI: 68.1 ± 9.7	61 (61)	NA	SCr↑≥50% baseline within 48 h after CPB	ELISA	–80
Gul et al. [43]	Turkey	51	15 (29.41)	CIN adults	AKI: 65.5 ± 11.1 Non-AKI: 61.5 ± 10.2	35 (68.6)	AKI: 29.0 (0.5–47.2); non-AKI: 17.5 (1.5–44.0)	SCr > 0.5 mg/dl or >25% within 72 h	ELISA	–80
Xin et al. [41]	China	33	9 (27.27)	Adults went through cardiac surgery	AKI: 37.04 ± 20.21; non-AKI: 39.56 ± 21.94	18 (54.5)	AKI: 67.89 ± 12.84; non-AKI: 68.94 ± 18.32^d^	AKIN criteria	ELISA	–80
Liangos et al. [28]	USA	103	13 (12.6)	Adults undergoing on-pump cardiac surgery	68 ± 11	74 (72)	NA	SCr↑≥50% baseline within the first 72 h after CPB	ELISA	–80
Liang et al. [45]	China	122	30 (24.59)	Adults went through CPB	AKI: 30 (24–46) Non-AKI: 30 (24–44)	NA	NA	RIFLE criteria	ELISA	–80
Endre et al. [29]	Australia	523	147 (28.1)	ICU adults	60 ± 17	367 (70.2)	73 ± 340^a^	SCr↑ >0.3 mg/dl or ≥50% baseline, AKIN48 or RIFLE24 criteria	ELISA	–80
Parikh et al. [30,31]	USA	311	53 (17)	Pediatric patients with congenital cardiac lesions surgery	3.8 ± 4.5	171 (55)	NA	Receipt of acute dialysis or SCr double baseline. RIFLE R or AKIN stage 2	ELISA	–80
Parikh et al. [30,31]	USA	1219	60 (4.9)	Cardiac surgery adults at high risk for AKI	71 ± 10	826 (68)	NA	Receipt of acute dialysis or SCr double baseline. RIFLE R or AKIN stage 2	ELISA	–80
Doi et al. [42]	Japan	339	131 (38.64)	ICU adults	NA	223 (65.8)	NA	RIFLE criteria	ELISA	–80
Chen et al. [32]	Taiwan, China	151	43 (28.7)	CCU adults	66 ± 1	113 (75)	71 ± 5	AKIN criteria	ELISA	–80
Nickolas et al. [46]	USA	1635	855 (52.3)	Adults in emergency department	NA	NA	NA	iAKI criteria	ELISA	–80
Torregrosa et al. [47]	Spain	135	101 (74.8)	CIN or cardiac surgery adults	NA	NA	NA	RIFLE criteria	ELISA	–80
Li et al. [33]	China	62	11 (17.7)	Non-septic critically ill neonates	34.1 ± 3.2^b^	34 (54.8)	NA	SCr > 1.5 mg/dl within first 3 days; after first 3 days, eCCl↓ ≥25% baseline. Modified pediatric RIFLE	ELISA	–80
Sirota et al. [34]	USA	40	7 (17.5)	Patients who underwent a first-time orthotopic liver transplantation	56.1 ± 6.8	27 (67.5)	AKI: 0 (0–18.57) Non-AKI: 0 (0–200.10)	SCr↑≥50% baseline, RIFLE and AKIN criteria	ELISA	–80
Zheng et al. [35]	China	58	29 (50)	Children with CHD or undergoing CPB surgery	Non-AKI: 11.4 (2.2–47.0) AKI: 5.9 (0.6–44.5)^c^	39 (67.2)	AKI: 17.6 (7.2–41.5) Non-AKI: 7.9 (3.8–23.1)	KDIGO criteria	ELISA	–80
Morales-Buenrostro et al. [36]	Mexico	37	17 (45.95)	Critically ill patients in ICU and exhibited two or more organ failures	51.6 ± 20.2	20 (54.1)	NA	AKIN criteria	ELISA	–80
Nisula et al. [37]	Finland	1439	268 (18.62)	Critically ill patients in ICU	63 (50–73)	920 (63.9)	NA	KDIGO criteria	ELISA	–80
Ren et al. [38]	China	58	11 (18.97)	Burn patients	Non-AKI: 39.1 ± 17.7 AKI: 33.4 ± 11.3	46 (79.3)	AKI: 9.64 ± 2.43 Non-AKI: 6.15 ± 2.57	KDIGO criteria	ELISA	–80
Wybraniec et al. [39]	Poland	95	9 (9.47)	Adults underwent CA/PCI	65 (59–71)	66 (69.5)	AKI: 119.8 Non-AKI: 81.2	SCr↑ >0.3 mg/dl or ≥50% baseline at 48 h after procedure	ELISA	–80
Hayashi et al. [40]	Japan	542	67 (12.2)	Patients undergoing elective open surgery for non-ruptured AAA	Non-AKI: 57 ± 17 AKI: 52 ± 18	NA	NA	KDIGO criteria	ELISA	–80
Al-Saegh et al. [50]	Iraq	78	30 (38.46)	Patients in ICU	Non-AKI: 51.1 ± 15.9 AKI: 53.8 ± 20.4	35 (44.87)	49.24 ± 40.2	KDIGO criteria	ELISA	–80
Tan et al. [49]	China	157	36 (22.93)	Patients with urosepsis after ureteroscopic lithotripsy	Non-AKI: 49.84 ± 11.36 AKI: 52.83 ± 10.21	14 (38.9)	Non-AKI: 12.30 ± 5.45 AKI: 13.92 ± 6.81	KDIGO criteria	ELISA	–80

AKI: acute kidney injury; IL: interleukin; ARDS: acute respiratory distress syndrome; ALI: acute lung injury; SCr: serum creatinine; ELISA: enzyme-linked immunosorbent assay; PICU: pediatric intensive care unit; CIN: contrast-induced nephropathy; RIFLE: risk, injury, loss, and end-stage renal disease; CPB: cardiopulmonary bypass; ICU: intensive care unit; AKIN: acute kidney injury network; CCU: coronary care unit; eCCl: estimated creatinine clearance; CHD: congenital heart disease; KDIGO: Kidney Disease: Improving Global Outcomes; CA/PCI: coronary angiography/percutaneous coronary interventions; AAA: abdominal aortic aneurysm; NA: not available.

^a^ (pg/ml)/mmol/l Cr.

^b^ Gestational age, weeks.

^c^ Months.

^d^ μmol/l.

### 3.2. Quality assessment and publication bias

**Figure 2.**
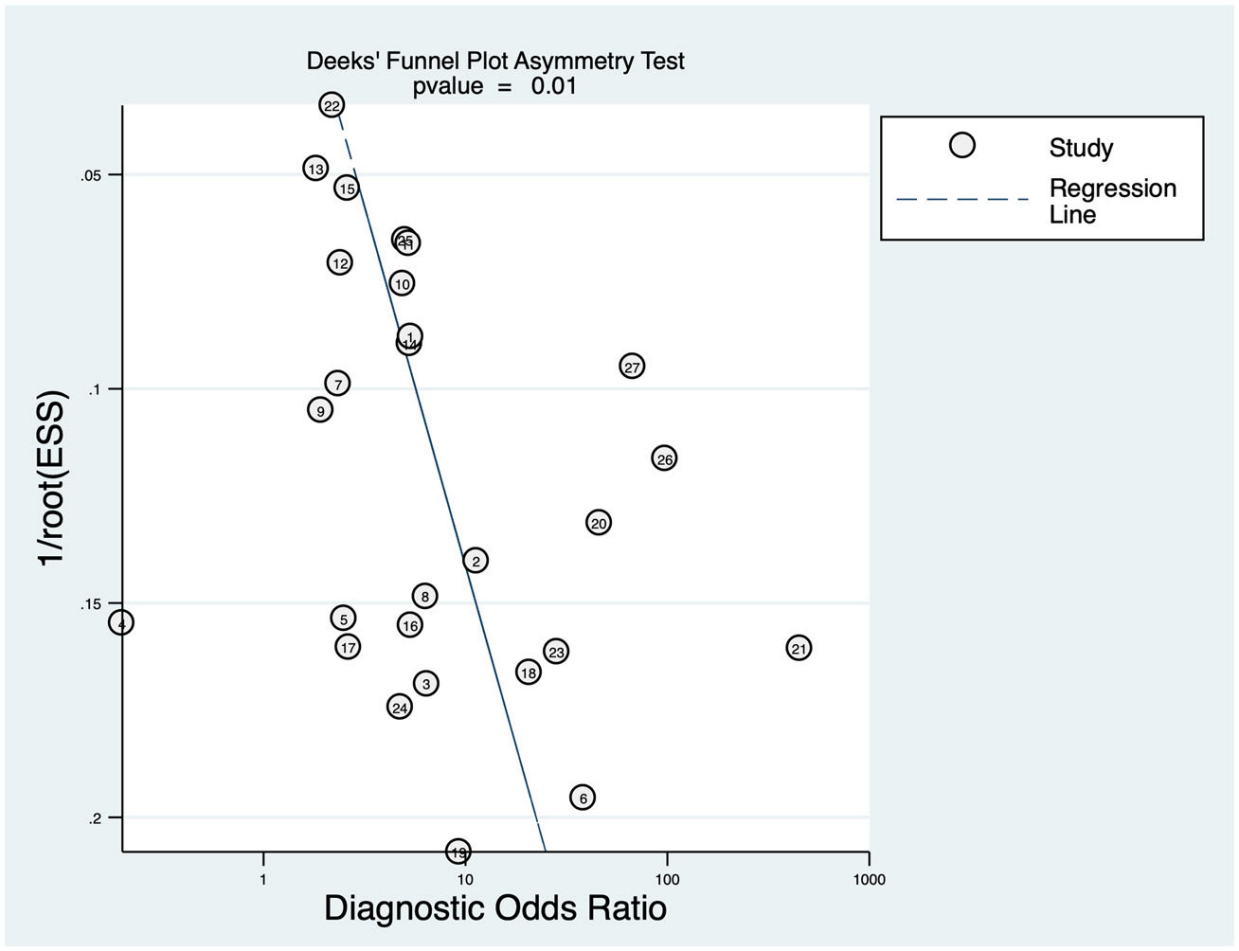
Deek’s funnel plot asymmetry test.

The methodological quality of the studies according to the QUADAS-2 is shown in Table 2. All of the domains were considered to be at low or unclear risk. Deek’s funnel plots are shown in Figure 2 with a *p* value of 0.01, indicating a statistically significant publication bias.

**Table 2.** Quality assessment of individual studies.

Study	Risk of bias				Applicability concerns		
	Patient selection	Index test	Reference standard	Flow and timing	Patient selection	Index test	Reference standard
Parikh et al. [26]	Low	Low	Low	Low	Low	Low	Low
Parikh et al. [20]	Low	Low	Low	Low	Low	Low	Low
Washburn et al. [27]	Low	Low	Low	Low	Low	Low	Low
Ling et al. [48]	Low	Low	Low	Low	Low	Low	Low
Haase et al. [44]	Low	Low	Low	Low	Low	Low	Low
Gul et al. [43]	Low	Low	Low	Low	Low	Low	Low
Xin et al. [41]	Low	Low	Low	Low	Low	Low	Low
Liangos et al. [28]	Low	Low	Unclear	Low	Low	Low	Unclear
Liang et al. [45]	Low	Low	Low	Low	Low	Low	Low
Endre et al. [29]	Low	Low	Low	Unclear	Low	Low	Low
Parikh et al. [30,31]	Low	Low	Low	Low	Low	Low	Low
Parikh et al. [30,31]	Low	Low	Low	Low	Low	Low	Low
Doi et al. [42]	Low	Low	Low	Low	Low	Low	Low
Chen et al. [32]	Low	Low	Unclear	Low	Low	Low	Unclear
Nickolas et al. [46]	Low	Low	Low	Low	Low	Low	Low
Torregrosa et al. [47]	Low	Low	Low	Low	Low	Low	Low
Li et al. [33]	Low	Low	Low	Low	Low	Low	Low
Sirota et al. [34]	Low	Unclear	Low	Low	Low	Low	Low
Zheng et al. [35]	Low	Low	Unclear	Low	Low	Low	Unclear
Morales (2014)	Low	Low	Low	Low	Low	Low	Low
Nisula et al. [37]	Low	Unclear	Low	Low	Low	Low	Low
Ren et al. [38]	Low	Low	Low	Low	Low	Low	Low
Wybraniec et al. [39]	Low	Unclear	Unclear	Low	Low	Unclear	Unclear
Hayashi et al. [40]	Low	Low	Low	Low	Low	Low	Low
Al-Saegh et al. [50]	Low	Low	Unclear	Low	Low	Low	Low
Tan et al. [49]	Low	Low	Low	Low	Low	Low	Low

The table summarizes the risk of bias and applicability concerns.

### 3.3. Data synthesis

**Figure 3.**
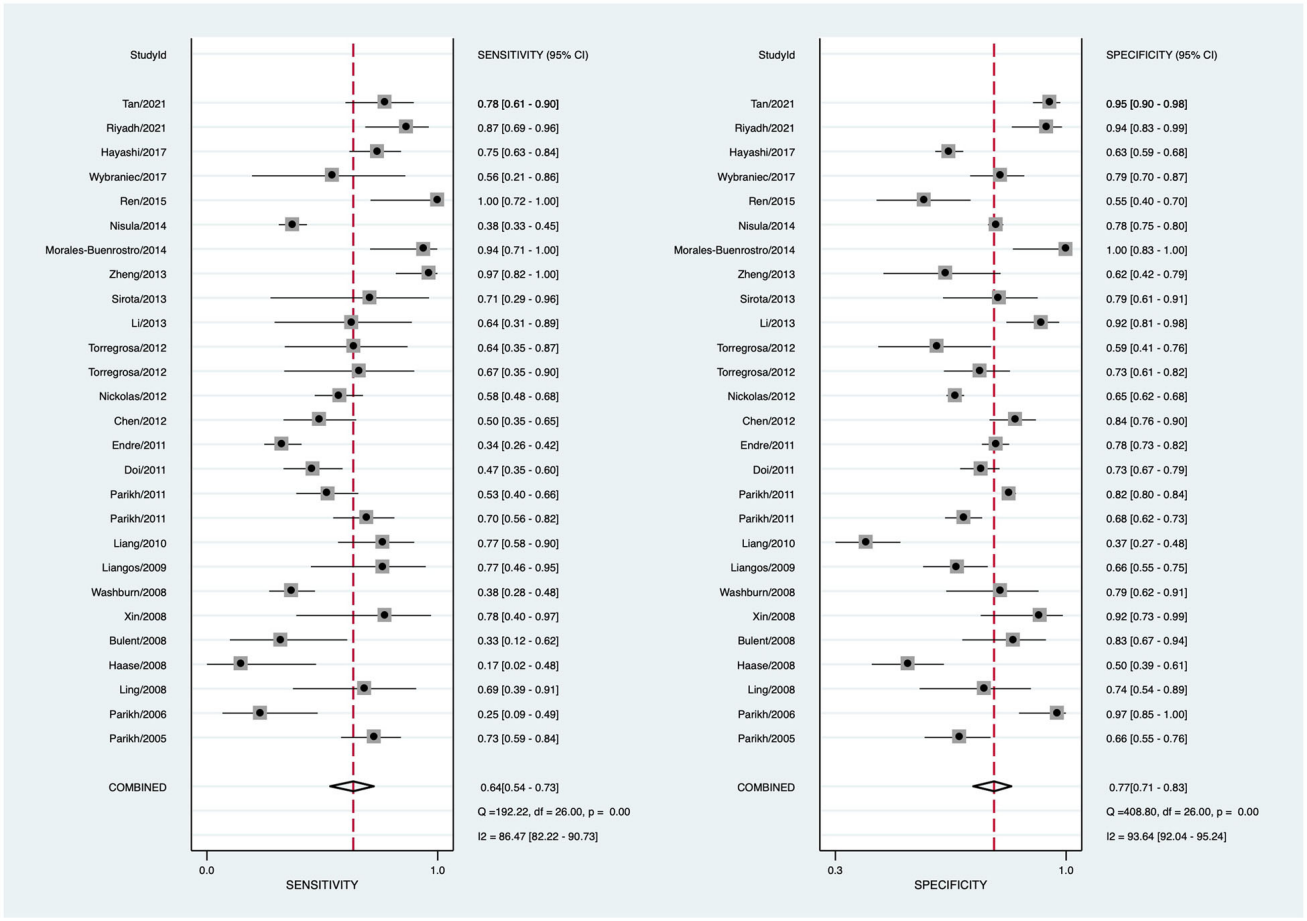
Forest plots of the pooled sensitivity and specificity of urinary IL-18 in predicting acute kidney injury across all settings.

**Figure 4.**
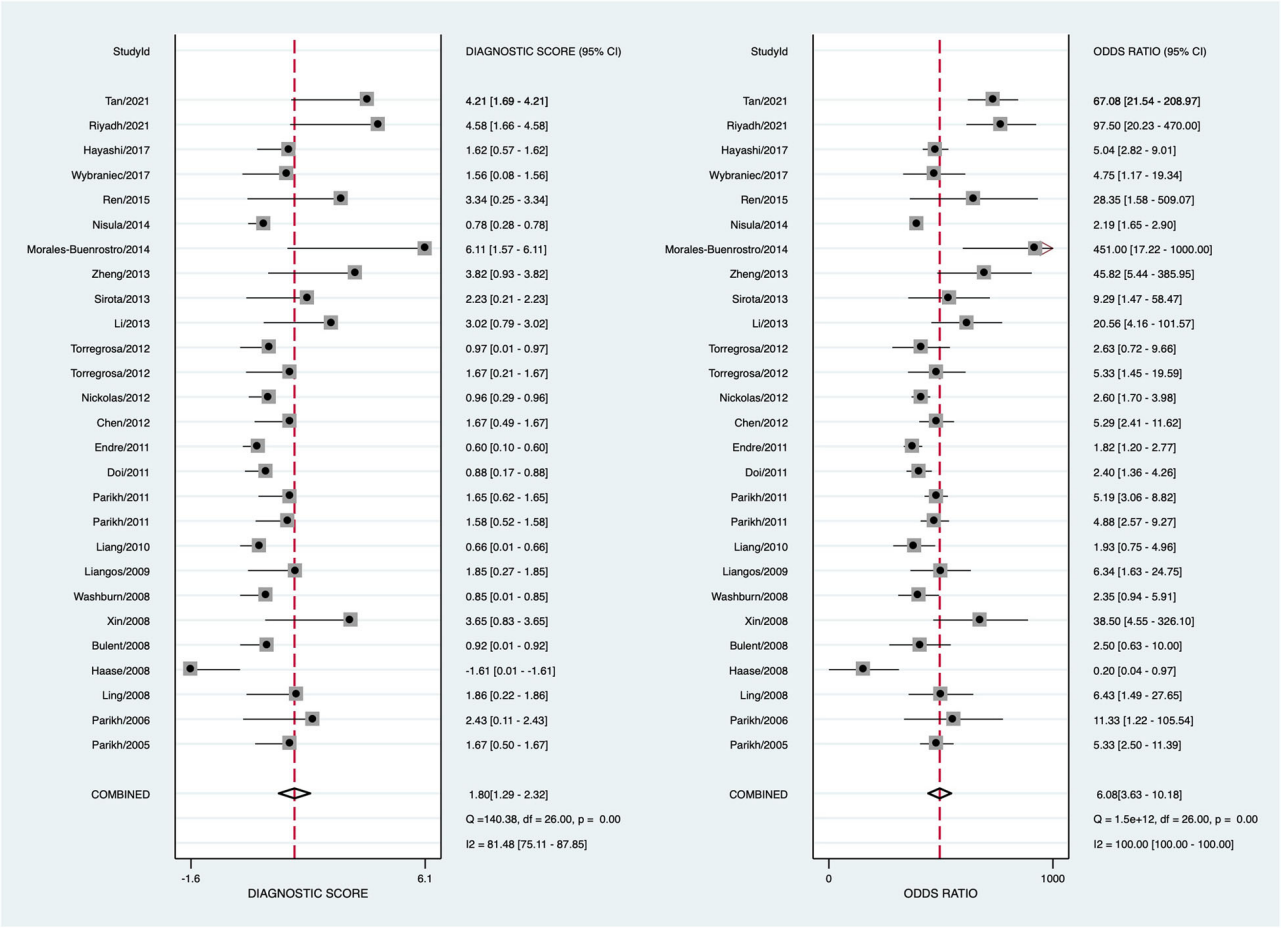
Forest plot of the pooled diagnostic odds ratio of urinary IL-18 in predicting acute kidney injury across all settings.

**Figure 5.**
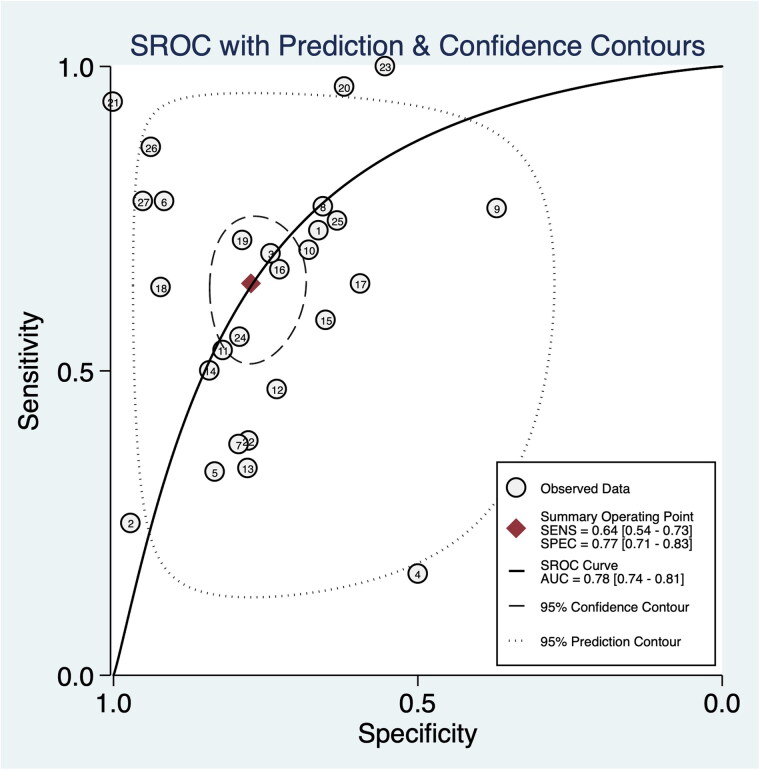
Hierarchical summary receiver operating characteristic (SROC) plots of urinary IL-18 to predict acute kidney injury across all settings.

Data in the 26 eligible studies were extracted and shown in Table 3, including TP, FN, FP, TN, the time of obtaining specimen, the assess time, the optimal cutoff value for urinary IL-18 with their sensitivity, specificity, and AUROC (95%CI). The estimated sensitivity and specificity values of uIL-18 in the diagnosis of AKI were 0.64 (95%CI: 0.54–0.73) and 0.77 (95%CI: 0.71–0.83), respectively (Figure 3). There was a substantial heterogeneity both in sensitivity and specificity, according to the *I*^2^ value of 86.47% and 93.64%, respectively. DOR was 6.08 (95%CI: 3.63–10.18) and shown in Figure 4; PLR and NLR were 2.82 (95%CI: 2.10–3.78) and 0.46 (95%CI: 0.36–0.61), respectively. The summary receiver operating characteristic (SROC) plot suggested that the efficiency of urinary IL-18 in diagnosing AKI was considerable, with the AUC of 0.78 (95%CI: 0.74–0.81) (Figure 5).

**Table 3.** Performance of IL-18 for AKI diagnosis in studies included in the meta-analysis.

Study	Time of obtaining specimen	TP	FP	FN	TN	Cutoff value (pg/ml)	Sensitivity (%)	Specificity (%)	AUROC (95%CI)	Assess time (h)
Parikh et al. [26]	Day 0, 1, and 3	38	29	14	57	25	74	66	0.731	24
Parikh et al. [20]	Every 2 h for the first 12 h and then once every 12 h	5	1	15	34	50	25	97	0.61	4
		10	2	10	33	50	50	94	0.75	12
		12	4	8	31	20	60	89	0.73	24
Washburn et al. [27]	2 PM each day	39	7	64	27	75	38	78	0.54 (0.31–0.77)	24
		55	10	48	24	75	53	71	0.61 (0.43–0.78)	48
Ling et al. [48]	24 h after CA	9	7	4	20	15.8	69	74	0.75 (0.58–0.92)	24
Haase et al. [44]	6 h after CPB	2	40	10	40	150	50	50	0.53 (0.38–0.68)	6
Gul et al. [43]	24 h after CA/PCI	5	6	10	30	0.29	33	83	0.52 (0.37–0.66)	24
Xin et al. [41]	2 h after cardiac surgery	7	2	2	22	2200	78	91	0.89	2
Liangos et al. [28]	2 h after CPB	10	31	3	59	92	75	66	0.66 (0.49–0.83)	2
Liang et al. [45]	6 h after CPB	13	16	17	76	60^a^	43	83	0.61 (0.52–0.70)	6
	12h after CPB	23	58	7	34	50^a^	77	37	0.62 (0.53–0.70)	12
Endre et al. [29]	Admission	50	83	97	293	36^b^	34	78	0.62 (0.56–0.67)	0
Parikh et al. [30,31]	Admission	37	83	16	175	125	69	68	0.72 (0.64–0.80)	48
Parikh et al. [30,31]	Admission, every 6 h	32	209	28	950	60	54	82	0.74 (0.66–0.81)	48
Doi et al. [42]	Within 12 h of ICU admission	31	56	35	152	133.8	47	73	0.59 (0.51–0.67)	12
Chen et al. [32]	Admission	22	17	22	90	70	50	84	0.621 (0.504–0.738)	48
Nickolas et al. [46]	Within 12 h of ICU or emergency department admission	56	398	40	740	36	58	65	0.64 (0.57–0.70)	12
Torregrosa et al. [47]	12 h after CA	8	21	4	56	202	67	73	0.73 (0.57–0.89)	24
	12h after heart surgery	9	13	5	19	249	64	60	0.68 (0.49–0.86)	24
Li et al. [33]	48 h admission later	7	4	4	47	1800^c^	64	92	0.72 (0.52–0.93)	48
Sirota et al. [34]	24 h after orthotopic liver transplantation	5	7	2	26	NA	72	79	0.749	24
Zheng et al. [35]	0, 4, 6, 12, and 24 h after the initiation of CPB	28	11	1	18	49	96.6	62.1	0.835 (0.729–0.940)	4
Morales-Buenrostro et al. [36]	AKI: 3 days before and until 2 days after the diagnosis Non-AKI: 1, 5 and 10 days after admission	16	0	1	20	150	92	100	0.92	48
		15	1	2	19	120	88	95	0.93	24
Nisula et al. [37]	Admission and 24 h later	103	260	165	911	65	38.4	77.8	0.586 (0.546–0.627)	24
Ren et al. [38]	Admission and 48 h later	11	21	0	26	6.39	100	56	0.819 (0.714–0.923)	0
Wybraniec et al. [39]	Within 24 h before and 6 h after CA/ PCI	5	20	4	76	118.2	56	88	0.75	6
Hayashi et al. [40]	The start of anesthesia, the period of aortic cross-clamping, the period of aortic declamping, the end of the surgery, and on POD	50	175	17	300	18.5	75	63	0.72 (0.66–0.79)	0
Al-Saegh et al. [50]	24, 48, and 72 h after admitted in ICU	26	3	4	45	15.63	87.5	94.4	0.946	24
Tan et al. [49]	0, 4, 12, 24, and 48 h after ureteroscopic lithotripsy	28	6	8	115	26.44	78.6	94.7	0.881 (0.815–0.947)	12

IL: interleukin; AKI: acute kidney injury; TP: true positive; FP: false positive; FN: false negative; TN: true negative; AUROC: area under the receiver operating curve; CI: confidence interval; CA: coronary angiography; CPB: cardiopulmonary bypass; ICU: intensive care unit; CA/PCI: coronary angiography/percutaneous coronary interventions; NA: not available; POD: post-operative day.

^a^ ng/mg UCr.

^b^ (pg/ml)/mmol/l Cr.

^c^ pg/mg UCr.

### 3.4. Subgroup analysis

We used meta-regression to explore the source of heterogeneity (Supplemental Table 2). Age, predictive time, obtaining specimen, population settings, and AKI definition contributed to the heterogeneity of specificity while we failed exploring the source of heterogeneity of sensitivity.

Subgroup analysis based on the patients age, predictive time, the time of obtaining specimen, population setting, publication date, AKI definition and population continents was conducted. As shown in Table 4, the diagnostic value of urinary IL-18 was higher in pediatrics group than in adult group, with a DOR of 7.33 (95%CI: 2.86–18.78) compared to 5.75 (95%CI: 3.17–10.42). The early AKI predictive time (≤12 h) was much more sensitive but less specific than predictive time >12 h. As for the time of specimen obtaining, obtaining specimen at admission time showed lower diagnostic value than obtaining specimen at other times, with a DOR of 4.12 (95%CI: 2.17–7.81) versus 7.20 (95%CI: 3.69–14.04). With regard to the population setting, patients underwent cardiac surgery showed increased sensitivity compared with other kinds of patients, while the DORs were 4.42 (95%CI: 2.39–8.16) and 8.11 (95%CI: 3.64–10.48). As for subgroup analysis stratified by the publication year before and after 2009 (halfway point), the pooled DOR of studies after 2009 (7.39, 95%CI: 4.01–13.63) was greater than those before 2009 (3.65, 95%CI: 1.48–9.00), with a higher AUC as well. Urinary IL-18 level had better diagnostic accuracy in studies using standardized AKI definition (DOR = 7.81, 95%CI: 7.81–7.81) than in those did not (DOR = 3.76, 95%CI: 1.82–7.77). The diagnostic value in population from Asia (DOR = 10.81, 95%CI: 4.92–23.73) was greater than those from North America (DOR = 4.59, 95%CI: 2.73–7.72) and other continents (DOR = 1.51, 95%CI: 0.51–4.59) and showed the highest AUC of 0.83.

**Table 4.** Subgroup analysis.

Factors	Subgroup	Sensitivity (95%CI)	Specificity (95%CI)	+LR (95%CI)	–LR (95%CI)	DOR (95%CI)	AUC (95%CI)
All studies (27)		0.64 (0.59–0.73)	0.77 (0.71–0.83)	2.82 (2.10–3.78)	0.46 (0.36–0.61)	6.08 (3.63–10.18)	0.78 (0.74–0.81)
*I*^2^ (%)		86.47	93.64	77.92	87.21	100	
Age	Adults (21)	0.64 (0.54–0.73)	0.76 (0.69–0.82)	2.69 (1.92–3.77)	0.47 (0.35–0.63)	5.75 (3.17–10.42)	0.77 (0.73–0.80)
	*I*^2^ (%)	85.25	94.34	79.64	87.50	100.00	
	Pediatrics (5)	0.61 (0.31–0.84)	0.83 (0.66–0.92)	3.5 (1.98–6.18)	0.48 (0.25–0.93)	7.33 (2.86–18.78)	0.81 (0.78–0.85)
	*I*^2^ (%)	92.32	87.35	19.14	87.32	95.43	
Predictive time	≤12 h (13)	0.69 (0.53–0.81)	0.73 (0.60–0.82)	2.51 (1.66–3.81)	0.43 (0.27–0.68)	5.87 (2.68–12.85)	0.77 (0.73–0.80)
	*I*^2^ (%)	82.62	92.44	76.58	88.54	100.00	
	>12 h (13)	0.60 (0.47–0.71)	0.81 (0.75–0.86)	3.15 (2.15–4.62)	0.50 (0.36–0.69)	6.31 (3.21–12.39)	0.80 (0.76–0.83)
	*I*^2^ (%)	86.43	85.71	80.90	89.01	100	
Obtaining specimen	Admission (6)	0.58 (0.36–0.77)	0.75 (0.68–0.81)	2.32 (1.83–2.94)	0.56 (0.36–0.87)	4.12 (2.17–7.81)	0.75 (0.71–0.79)
	*I*^2^ (%)	87.63	88.32	45.91	77.60	98.43	
	Other times (20)	0.66 (0.56–0.76)	0.78 (0.70–0.85)	3.08 (2.07–4.57)	0.43 (0.31–0.59)	7.20 (3.69–14.04)	0.79 (0.75–0.82)
	*I*^2^ (%)	84.19	93.08	81.29	88.39	100.00	
Patients population	Cardiac surgery (12)	0.62 (0.48–0.75)	0.73 (0.62–0.81)	2.29 (1.63–3.21)	0.52 (0.37–0.73)	4.42 (2.39–8.16)	0.74 (0.69–0.77)
	*I*^2^ (%)	76.37	92.57	56.00	81.94	100.00	
	Other patients (14)	0.65 (0.52–0.77)	0.81 (0.73–0.87)	3.46 (2.16–5.52)	0.43 (0.29–0.63)	8.11 (3.64–18.10)	0.81 (0.77–0.84)
	*I*^2^ (%)	91.48	96.75	90.68	93.38	100	
Publication date	Before 2009 (including 2009) (8)	0.51 (0.34–0.68)	0.78 (0.65–0.87)	2.30 (1.29–4.11)	0.63 (0.43–0.92)	3.65 (1.48–9.00)	0.71 (0.67–0.75)
	*I*^2^ (%)	81.52	84.26	22.39	83.32	99.99	
	After 2009 (18)	0.69 (0.58–0.78)	0.77 (0.69–0.83)	2.99 (2.13–4.22)	0.41 (0.29–0.56)	7.39 (4.01–13.63)	0.80 (0.76–0.83)
	*I*^2^ (%)	88.58	95.65	87.44	90.04	100	
AKI definition	Standardized definition (RIFLE, AKIN, KDIGO) (17)	0.69 (0.69–0.69)	0.78 (0.78–0.78)	3.09 (3.09–3.09)	0.39 (0.39–0.39)	7.81 (7.81–7.81)	0.80 (0.77–0.84)
	*I*^2^ (%)	89.90	94.23	85.98	90.19	100	
	Non-standardized definition (9)	0.53 (0.39–0.67)	0.77 (0.65–0.86)	2.30 (1.43–3.68)	0.61 (0.45–0.83)	3.76 (1.82–7.77)	0.70 (0.66–0.74)
	*I*^2^ (%)	72.90	89.64	42.09	85.29	100	
Continents	Asia (12)	0.74 (0.60–0.84)	0.79 (0.67–0.88)	3.56 (2.20–5.76)	0.33 (0.21–0.52)	10.81 (4.92–23.73)	0.83 (0.80–0.86)
	*I*^2^ (%)	82.22	94.66	88.19	85.65	100	
	North America (11)	0.58 (0.45–0.70)	0.77 (0.70–0.83)	2.51 (1.18–3.36)	0.55 (0.41–0.72)	4.59 (2.73–7.72)	0.76 (0.72–0.79)
	*I*^2^ (%)	85.84	93.53	34.73	80.45	100	
	Other (3)	0.43 (0.24–0.64)	0.67 (0.55–0.78)	1.29 (0.66–2.53)	0.86 (0.55–1.33)	1.51 (0.50–4.59)	0.60 (0.56–0.64)
	*I*^2^ (%)	79.84	89.55	72.25	84.56	99.61	

## 4. Discussion

Early prediction and diagnosis of AKI is of great importance to improve prognosis of patients. The existing diagnostic indicators including decreased urine volume and increased SCr had the shortcoming of time limitation and could not fully meet the clinical needs, leading to the delay of AKI diagnosis, thus showing a negative effect on the prognosis. Finding a more diagnosis measurement for AKI is urgent in clinical practice.

In this diagnosis meta-analysis, we conducted a comprehensive search to identify articles that evaluated the diagnostic performance of uIL-18 in predicting AKI. After screen and full-text review, a total of 26 studies with 7183 patients were included and relevant information was extracted. We found using uIL-18 in diagnosis AKI was considerable, with the estimated sensitivity, specificity, DOR, and AUC of 0.64 (95%CI: 0.59–0.73), 0.77 (95%CI: 0.71–0.83), 6.08 (95%CI: 3.63–10.18), and 0.78 (95%CI: 0.74–0.81), respectively. When DOR is greater than 1, a higher DOR indicates a better test performance [51]. Although this topic has been studied before by Lin et al. [52], it has been almost 10 years since the last publication, and they only enrolled 11 studies. Our study updated the included studies and demonstrated that uIL-18 showed a moderate diagnostic accuracy for AKI, while its sensitivity and specificity were poor, which is similar with previous results. In addition, we found that uIL-18 in pediatric patients was more effective in predicting AKI than in adults, reminding us the clinical potential in different population settings.

A potential explanation of this predictive value of uIL-18 for AKI maybe the relationship between IL-18 and inflammation. The expression of IL-18 could be upregulated as a part of cell inflammation damage by activating macrophages, promoting T cell differentiation and stimulating NK/T cell in releasing γ-interferon. The expression of IL-18 also could be observed in other cell lineages besides the renal tubule cells, including macrophages, lymphocytes, intestinal epithelial cells, and fibroblasts. In the acute phase of disease or in the state of systemic inflammatory response, the systemic expression of IL-18 could be upregulated, which may affect the relationship between urinary IL-18 and AKI, thus affecting the accuracy of diagnosis for AKI in sensitivity and specificity [53–55]. AKI is a complex clinical condition that may cannot be completely predicted by a single biomarker. It was believed that the combination of biomarker and clinical risk stratification for the diagnosis of AKI would be the trend of future research [56,57].

There was a significant heterogeneity among the included studies, that suggested the application of uIL-18 in predicting AKI might be limited. To fully evaluate the diagnostic value of uIL-18 for AKI, we conducted subgroup analysis based on the patients age, predictive time, the time of obtaining specimen, population setting, publication date, and AKI definition. The diagnostic value of urinary IL-18 was higher in pediatrics group than in adult group, with a DOR of 7.33 (95%CI: 2.86–18.78) compared to 5.75 (95%CI: 3.17–10.42). We hypothesized that this may be related to the significant comorbidities in adults, such as hypertension, atherosclerotic disease, diabetes mellitus, etc. These complex comorbidities were much more common in adults and may affect the concentrations of urinary IL-18 through inflammation pathway. In addition, due to the children and adolescents exhibit different physiological states in renal mutations, their kidneys undergo a process of growth and maturation, the risk factors associated with AKI and the timing of kidney injury remain unclear [58]. Both the metabolic capacity and compensatory ability of children and adolescents also varied from those of adults, which may contribute to the different kidney injury condition and urinary IL-18 levels as well [59,60]. Similar results also have been reported before, which reminds us the need of a broad panel of biomarkers [61]. In addition, there was no significant reduction of heterogeneity in these two subgroups, which may be due to the different cutoff values, different AKI definitions, different clinical setting, and varied patient number among the included studies. As for the predictive time, the predictive time ≤12 h group was much more sensitive but less specific than predictive time >12 h group, and there was no significant difference in its predictive value. Obtaining specimen at non-admission time showed higher diagnostic value than obtaining specimen at admission, with a DOR of 7.20 (95%CI: 3.69–14.04) compared to 4.12 (95%CI: 2.17–7.81), while the heterogeneity still cannot be ignored. With regard to the population setting, patients underwent cardiac surgery showed increased sensitivity compared with other kinds of patients, while the DORs were 4.42 (95%CI: 2.39–8.16) and 8.11 (95%CI: 3.64–18.10), respectively. We also conducted subgroup analysis stratified by the publication year before and after 2009 (half way point), the pooled DOR of studies after 2009 (7.39, 95%CI: 4.01–13.63) was greater than those before 2009 (3.65, 95%CI: 1.48–9.00), indicating the changing performance of uIL-18 may be based on time frame of care. Regarding AKI definition, subgroup analyses divided by those studies that used standardized definition (RIFLE, AKIN, and KDIGO) and those that did not were performed as well. We found that uIL-18 level had better diagnostic accuracy in studies using standardized AKI definition (DOR = 7.81, 95%CI: 7.81–7.81) than in those did not (DOR = 3.76, 95%CI: 1.82–7.77), which is useful in clinical management, since prevention strategies can be developed if AKI can be predicted in advance. Other factors, such as the cutoff value of urinary IL-18, the different conditions of patients may also be the source of heterogeneity, which cannot be further explored due to the limitations of the included study.

The results of this study suggested that the diagnostic accuracy of uIL-18 in predicting AKI was moderate, but we could not determine the ideal cutoff value for uIL-18 because the range of cutoff values included in the studies varied widely from 6.39 pg/ml to 125 pg/ml. In addition, there were three studies used IL-18 concentration standardized by urinary creatinine or absolute concentration. Given the factors we mentioned before, it is still difficult for us to determine a suggested cutoff point of uIL-18. At present, the consensus threshold of IL-18 for risk stratification for AKI has not been reached yet, while some investigators suggested a cutoff range of 100–500 pg/mg Cr [62]. Ralib et al. found that absolute concentrations showed the best predictive value for AKI at admission, while standardized concentrations performed a better prediction of subsequent development of AKI and death [63]. However, Waikar et al. suggested that normalized levels of biomarkers reflecting tubular injury could be influenced by dynamic changes in the UCr excretion rate when the GFR changed. Thus, the standardized concentrations might lead to an underestimation or overestimation [64]. Therefore, the recommended range of uIL-18 in predicting AKI remains controversial and further studies are needed.

Many biomarkers have been studied to evaluate their predictive value for AKI. An international conference proceeding including the AKI definition, staging and the potential biomarkers has been published before, and recommended 14 biomarkers for further research [65]. It was noting that there is only one FDA-approved biomarker product commercially available in the United States, which is tissue inhibitor of metalloproteinase-2 (TIMP-2) and insulin‐like growth factor‐binding protein 7 (IGFBP7). It has been promoted to predict AKI and presented the best performance over all the other biomarkers. In 2013, Kashani et al. recommended [TIMP‐2] × [IGFBP7] as a risk score for KDIGO‐2 and KDIGO‐3 stage AKI in a wide range of intensive care settings [66]. Previous studies have shown that the test is feasible in patients after cardiac surgery, especially in those at intermediate to high risk for AKI [67–69]. In a prospective cohort study enrolled 442 ICU patients, a negative [TIMP‐2] × [IGFBP7] (<0.3 ng/ml) versus positive results on admission was predictive of AKI (31.9% versus 68.10%, respectively; *p* < 0.001), and AKI stage 2 or 3 was higher in patients with a positive assay result (*p* = 0.026). The predictive ability of a positive assay decreased over time (within 12 h AUC = 0.74, 95%CI = 0.69–0.80; 48-h AUC = 0.70, 95%CI = 0.65–0.76; and between 48 h and seven days AUC = 0.40, 95%CI = 0.28–0.52), with the best performance for AKI stage 2 or 3 within 12 h (AUC = 0.82; 95%CI = 0.70–0.88) [70]. However, one of the potential limitations of this biomarker is the inability to distinguish between transient and persistent AKI. In addition, although there is evidence that using [TIMP‐2] × [IGFBP7] in combination with routine testing, including SCr and urine volume, within 12 h of patient evaluation in the general surgical ICU helps predict the progression of AKI, there is little substantive evidence that its use improves clinically important patient outcomes [71]. Neutrophil gelatinase-related lipid carrier protein (NGAL) is a member of the lipid carrier protein superfamily, which is expressed by neutrophils and various epithelial cells. It has been regarded as a proximal tubular injury-related protein and been widely studied for the early prediction of AKI. Zhang et al. reported NGAL was a valuable predictor of sepsis-related AKI and associated with the mortality [72]. Xie et al. further explored the different diagnostic performance of urinary and plasma NGAL, they found that urinary NGAL showed higher diagnostic value than plasma NGAL in predicting AKI, especially in predicting sepsis related AKI (plasma NGAL: AUC = 0.86, urinary NGAL: AUC = 0.90) [73]. Kidney injury molecule-1 (KIM-1) is a type I transmembrane glycoprotein with immunoglobulin and mucin domains, which is not expressed in normal kidney tissue or urine, but highly expressed after ischemic or toxic renal injury. Its urine excretion could increase 2–8 h after injury significantly, thus helping the early diagnosis of AKI [74]. In addition, elevated urinary KIM-1 has the highest specificity in the recognition of renal injury, which can distinguish prerenal AKI from acute tubular necrosis [75]. In this analysis, we found the diagnosis of AKI with urinary IL-18 showed a sensitivity of 0.64 (95%CI: 0.59–0.73) and a specificity of 0.77 (95%CI: 0.71–0.83), indicating a moderate predictive value. Since IL-18 is a proinflammatory cytokine and its measurement may also be influenced by many coexisting variables including endotoxemia, inflammation, and autoimmune diseases, its sensitivity and specificity could be affected. In another meta-analysis comparing different biomarkers for sepsis-associated AKI, Xie et al. found the rank of urinary KIM-1 > urinary NGAL > plasma NGAL > urinary IL-18 according to the SROC curve area, suggesting that urinary KIM-1 has the highest diagnostic value. However, since the small simple size in Xie et al.’s study, studies with larger sample size are still needed in clinical practice [73]. With the addition of biomarkers to routine AKI monitoring parameters, further studies are warranted to identify those most likely to benefit among critically ill patients.

The limitation of this study cannot be ignored. First, the number of included studies was small. Second, there was a significant heterogeneity among the included studies in terms of different population setting, age, predictive time, AKI definition, the time of obtaining specimen and cutoff value. Moreover, even though both meta-regression and subgroup analysis were conducted to explore the source of heterogeneity, the marked heterogeneity still existed. Another limitation was the publication bias, which is suggested by Deek’s funnel plots and cannot be altered through statistical methods. In addition, all the included studies used SCr or urine volume as the diagnostic criteria for AKI, which are not ideal indicators for the diagnosis of early kidney injury [76]. Radio-labeled tracer clearance or evidence of damage from renal biopsy maybe a better method to diagnosis AKI. But they are time-consuming, invasive, or radioactive, thus limiting their commonly application in routine clinical practice. Additionally, we did not register this study online before. Lastly, the predictive value of uIL-18 in the progression and prognosis of AKI is of great clinical significance as well, but it has not been discussed in this study. To sum up, current analysis based on very different clinical situation and patient populations, heterogeneity is inevitable with respect to AKI definition, AKI setting, time of specimen acquisition, and the predictive value of urinary IL-18 assessed by experimental groups. We tried to explore the sources of heterogeneity using meta-regression but failed, and the subgroup analysis did not reduce the heterogeneity significantly. Our findings are hypothesis generating but heterogeneity and publication bias limit use, thus, further studies are still needed.

## 5. Conclusions

Urinary IL-18 could be a relatively good biomarker for predicting AKI, especially in pediatric patients. However, further research and clinical settings are still needed to validate the potential application.

## Supplementary Material

Supplemental Material

## Data Availability

All data analyzed during this study are available in the public domain.
