# Supplementary material for: The value of urinary interleukin-18 in predicting acute kidney injury: a systematic review and meta-analysis
Source: Ren Fail. 2022 Oct 19;44(1):1717–31. doi: 10.1080/0886022X.2022.2133728 (PMC9586591; doi:10.1080/0886022X.2022.2133728)
Supplement: Supplemental Material [file IRNF_A_2133728_SM6008.pdf]

Supplemental Item 1. The detailed search strategy

PubMed:

((IL-18[Title/Abstract]) OR (interleukin 18[Title/Abstract])) AND ((acute kidney injury [Title/Abstract]) OR (acute renal failure[Title/Abstract]) OR (AKI[Title/Abstract]) OR (ARF[Title/Abstract]))

Medline:

1. "Acute Kidney Injury" [MeSH]

2. exp kidney failure, acute/ or exp kidney tubular necrosis, acute/

3. (acute kidney failure or acute renal failure).tw.

4. (acute kidney injur$ or acute renal injur$).tw.

5. (acute kidney insufficie$ or acute renal insufficie$).tw.

6. acute tubular necrosis.tw.

7. (ARF or AKF or ATN).tw.

8. or/1-7

9. "Interleukin-18" [MeSH]

10. Interleukin-18.tw.

11. IL-18.tw.

12. or/9-11 13. and/8,12

Embase:

1. acute kidney failure/ or acute kidney tubule necrosis/

2. (acute renal insufficien$ or acute kidney insufficien$).tw.

3. (acute kidney failur$ or acute renal failure$).tw.

4. (acute kidney injur$ or acute renal injur$).tw.

5. (ARF or AKI or ATN).tw.

6. acute tubular necrosis.tw

7. or/1-6

8. Interleukin-18.tw.

9. IL-18.tw.

10. or/8-9

11. and/7,10

Cochrane Library:

("IL-18" or "interleukin 18") and ("acute kidney injury" or "acute renal failure" or "AKI" or "ARF")

Supplemental Table 1. The data for kappa statistic.

Reviewer 1	Reviewer 2		Total
	Exclude	Include	
**Exclude**	28	2	30
**Include**	3	23	26
**Total**	31	25	56

Supplemental Table 2. Univariable Meta-regression.

Factors		Sensitivity	95%CI	P value	Specificity	95%CI	P value
Age	Adults (21)	0.65	0.55-0.75	0.75	0.76	0.69-0.83	0.02
	Pediatrics (5)	0.62	0.40-0.83		0.83	0.71-0.94	
Predictive time	≤12h (13)	0.68	0.56-0.81	0.80	0.72	0.63-0.81	0.00
	>12h (13)	0.60	0.47-0.73		0.82	0.75-0.88	
Obtaining specimen	Admission (6)	0.57	0.38-0.76	0.23	0.75	0.63-0.88	0.04
	Other times (20)	0.66	0.56-0.76		0.78	0.71-0.85	
Patients population	Cardiac surgery (12)	0.62	0.49-0.76	0.30	0.73	0.64-0.82	0.00
	Other patients (14)	0.66	0.53-0.78		0.81	0.74-0.88	
Publication Date	Before 2009 (8)	0.51	0.33-0.69	0.07	0.78	0.67-0.89	0.06
	After 2009 (18)	0.69	0.59-0.79		0.77	0.70-0.84	
AKI definition	Standardized definition (17)	0.69	0.59-0.79	0.08	0.77	0.70-0.85	0.03
	Non-standardized definition (9)	0.53	0.36-0.70		0.77	0.66-0.87	
Continents	Asia	0.73	0.62-0.84	0.91	0.79	0.70-0.88	0.03
	North America	0.59	0.46-0.73		0.79	0.70-0.88	
	Other continents	0.43	0.23-0.65		0.66	0.35-0.96	
